# Animal Models of Emerging Tick-Borne Phleboviruses: Determining Target Cells in a Lethal Model of SFTSV Infection

**DOI:** 10.3389/fmicb.2017.00104

**Published:** 2017-01-30

**Authors:** Keita Matsuno, Yasuko Orba, Kimberly Maede-White, Dana Scott, Friederike Feldmann, Mifang Liang, Hideki Ebihara

**Affiliations:** ^1^Molecular Virology and Host-Pathogen Interaction Unit, Laboratory of Virology, Division of Intramural Research, National Institute of Allergy and Infectious Diseases, National Institutes of Health, Rocky Mountain Laboratories, HamiltonMT, USA; ^2^Laboratory of Microbiology, Graduate School of Veterinary Medicine, Hokkaido UniversitySapporo, Japan; ^3^Global Station for Zoonosis Control, Global Institution for Collaborative Research and Education, Hokkaido UniversitySapporo, Japan; ^4^Division of Molecular Pathobiology, Research Center for Zoonosis Control, Hokkaido UniversitySapporo, Japan; ^5^Rocky Mountain Veterinary Branch, Rocky Mountain Laboratories, Division of Intramural Research, National Institute of Allergy and Infectious Diseases, National Institutes of Health, HamiltonMT, USA; ^6^NHFPC Key Laboratory for Medical Virology, National Institute for Viral Disease Control and Prevention, China CDCBeijing, China; ^7^Department of Molecular Medicine, Mayo Clinic, RochesterMN, USA

**Keywords:** aged mouse, disease modeling, heartland virus, immunocompromised mouse, mouse, nonhuman primate, severe fever with thrombocytopenia syndrome virus

## Abstract

The pathogenesis of clinical manifestations caused by newly emerging tick-borne phleboviruses [i.e., Severe fever with thrombocytopenia syndrome virus (SFTSV) and Heartland virus (HRTV)], such as severe thrombocytopenia and lymphocytopenia, are not yet fully understood. In the present study, to establish an animal model mimicking the profile of fatal human cases, we examined the susceptibilities of adult mice from 12 strains, aged mice from two strains, and cynomolgus macaques to SFTSV and/or HRTV infections. However, none of these immunocompetent animals developed lethal diseases after infection with SFTSV or HRTV. Thus, we tested a lethal animal model of SFTSV infection using interferon-α/β receptor knock-out (IFNAR^-/-^) mice to identify the target cell(s) of virus infection, as well as lesions that are potentially associated with hematological changes. IbaI-positive macrophages and Pax5-positive immature B cells overlapped with SFTSV-positive cells in the spleen and lymph nodes of IFNAR^-/-^ mice, and IbaI-SFTSV-double positive cells were also observed in the liver and kidney, thereby suggesting crucial roles for macrophages in the pathogenesis of SFTSV infection in mice. In the mandibular lymph nodes and spleens of infected mice, we observed extensive necrosis comprising B220-positive B cells, which may be associated with severe lymphocytopenia. The results of this study suggest a resemblance between the IFNAR^-/-^ mouse model and lethal infections in humans, as well as roles for multiple cells during pathogenesis in mice.

## Introduction

Emerging tick-borne pathogens in the genus *Phlebovirus* in the family *Bunyaviridae*, such as Severe fever with thrombocytopenia syndrome (SFTS) virus (SFTSV) and Heartland virus (HRTV), cause a severe, often fatal, febrile illness in humans (Yu et al., 2011; McMullan et al., 2012). SFTS cases have been identified in East Asian countries [i.e., China, Japan (Takahashi et al., 2014), and South Korea (Yun et al., 2016)], whereas HRTV infections have only been reported in the USA (Stubbs and Steele, 2014). In general, SFTS begins with a high fever (the fever stage) with marked thrombocytopenia, leukocytopenia, and a high serum viral load, followed by the multi-organ dysfunction (MOD) stage, which might be a consequence of systemic inflammatory response syndrome and disseminated intravascular coagulation (DIC) (Matsuno et al., 2014). The serum viral load, which is considered to be a prognostic marker associated with a fatal outcome (Gai et al., 2012; Li, 2013), remains high during the MOD stage but decreases in the convalescent patients. In addition to the serum viral load, thrombocytopenia is a characteristic of this disease that determines the fate of patients. However, the connection(s) between thrombocytopenia/lymphocytopenia and virus replication is still unclear (Hiraki et al., 2014; Takahashi et al., 2014).

Developing an animal model of severe/fatal SFTS in humans is crucial for understanding the pathogenesis of SFTSV infection and the immune response, which could facilitate the development of medical countermeasures such as a vaccine and therapeutics to combat SFTSV. Experimental infection with SFTSV causes non-fatal mild disease with a moderate decrease in platelets in immunocompetent laboratory animals (Chen et al., 2012; Jin et al., 2012, 2015), whereas immunocompromised interferon α/β receptor-knock-out (IFNAR^-/-^) mice exhibit 100% lethality after SFTSV infection (Liu et al., 2014). The IFNAR^-/-^ mouse model has a disadvantage because of lacking the initial antiviral response in infected animals, but the lethal IFNAR^-/-^ mouse model is useful for studying the pathogenesis of severe and fatal forms of SFTS and also for developing vaccines or antiviral drugs (Tani et al., 2015). Furthermore, fibroblastic reticular cells have been identified as the targets of SFTSV infection in the IFNR^-/-^ mice (Liu et al., 2014), while other cell types, such as monocytic or blastic cells have been suggested as the targets of SFTSV infection in humans based on hematological and/or histopathological studies (Yu et al., 2011; Takahashi et al., 2014; Peng et al., 2016). The differences and similarities between the mouse model and human cases should be studied further for understanding of a crucial step of the pathogenesis.

In the present study, we first examined the susceptibilities to SFTSV in 12 different immunocompetent inbred/outbred mouse strains, aged mice, IFNAR^-/-^ mice, and a nonhuman primate (cynomolgus macaque) in order to establish an animal model for SFTS. Finally, we pathologically examined the lethal IFNAR^-/-^ mouse model to determine its histopathological resemblance to fatal human SFTS infections.

## Materials and Methods

### Virus and Cells

Severe fever with thrombocytopenia syndrome virus strain SD4 was provided by the Chinese Center for Disease Control and Prevention and HRTV strain Mo4 was kindly provided by the World Reference Center for Emerging Viruses and Arboviruses (WRCEVA) from the arthropod-borne virus reference collection at the University of Texas Medical Branch (UTMB). These strains were propagated in a human hepatocyte cell line, i.e., human hepatocellular carcinoma cells (Huh7), for 5 days and the supernatant was centrifuged twice to remove any debris. The Huh7 cell line was kindly provided by Dr Yoshiharu Matsuura, Osaka University, and it was maintained in Dulbecco’s Modified Eagle Medium supplemented with 2% fetal calf serum, 2 mM L-glutamine, 50 U/ml penicillin, 50 μg/ml streptomycin (Life Technologies), and 10 μg/ml MycoKill AB (GE Healthcare). The virus infectivity titers in blood were determined as the median tissue culture infectious dose (TCID_50_) detected by using an indirect immunofluorescent antibody assay with mouse immune ascitic fluids generated against SFTSV or HRTV, which were provided by Dr. Robert Tesh, UTMB (Matsuno et al., 2013).

### Animal Study and Sample Collection

Groups of four 6- to 12-week-old mice from inbred strains (i.e., 129S1/svlmJ, A/J, C57BL/6J, CAST/EiJ, DBA/1J, DBA/2J, FVB/NJ, NZBWF1/J, and SIL/J), recombinant inbred strains (i.e., BXD68/RwwJ and BXD34/TyJ), an outbred strain (i.e., ICR CD-1) (obtained from Jackson Laboratory), IFNAR^-/-^ C57BL/6 (breeding pairs kindly provided by Dr. Genhong Cheng, University of California Los Angeles), and aged (10 to >20 months) 129S1/svlmJ and C57BL/6J strains (obtained from Jackson Laboratory and kept in-house) were inoculated with a high dose (10^5^ TCID_50_/animal) or a low dose (10^2^ TCID_50_/animal) of SFTSV SD4 intradermally (i.d.), intraperitoneally (i.p.), intramuscularly (i.m.), or subcutaneously (s.c.). The animals were monitored for 14 days after challenge. Animals that reached the humane endpoint were euthanized and terminally bled by cardiac puncture. To determine the median mouse lethal dose (MLD_50_), groups of four IFNAR^-/-^ C57BL/6 mice were infected with serial 10-fold dilutions of SFTSV SD4 and observed for 2 weeks.

Two groups of two cynomolgus macaques (2 to 3 kg) were inoculated s.c. with 10^6^ TCID_50_ of SFTSV SD4 or HRTV Mo4. At 1, 3, 5, 8, 11, and 14 days post inoculation (dpi), blood was drawn and clinical exams were performed on the anesthetized animals.

### Hematology

Hematological parameters were analyzed using EDTA-treated whole blood with a HemaVet 950FS1 laser-based hematology analyzer (Drew Scientific). The parameters of infected IFNAR^-/-^ mice and uninfected mice euthanized were compared statistically by Mann-Whitney test on GraphPad Prism v6.0h (GraphPad Software).

### Histopathology and Immunohistochemistry

Tissues fixed with neutral-buffered formalin (10% v/v) were processed and embedded in paraffin according to standard procedures. The embedded tissues were sectioned at 5 μm and dried overnight at 42°C before staining with hematoxylin and eosin. To detect the viral antigen by immunohistochemistry, a rabbit polyclonal antiserum against SFTSV N protein (kindly provided by Dr. Shigeru Morikawa, National Institute of Infectious Diseases, Japan) was used as the primary antibody. Different types of cells were identified immunohistochemically as follows: white blood cells [CD45R (B220)], immature B cells (Pax5), T cells (CD3e), macrophages (IbaI), and reticular cells (gp36), which were stained with Rat anti-CD45R (BD Biosciences), Rabbit mAb PAX5 (Cell Signaling), Goat anti-CD3-𝜀 (Santa Cruz), Rabbit anti-Iba-1 (Wako), and Hamster anti-Podoplanin (Novusbio), respectively. Each antigen was visualized with Envision++ system HRP Rabbit (DAKO), Simple stain AP Rabbit, Simple Stain MAX PO Rat (Nichirei), ImmPress HRP anti-goat IgG (Vector), or Biotinylated anti-Hamster IgG (Vector) with SAB-PO (Nichirei) in an appropriate combination according to the manufacturer’s protocol.

### Biosafety Statement

All infectious work with SFTSV or HRTV was performed in a high containment facility at the Rocky Mountain Laboratories (RML), Division of Intramural Research (DIR), National Institute of Allergy and Infectious Diseases (NIAID), National Institutes of Health (NIH). The work was approved by the RML Institutional Biosafety Committee (IBC) at biosafety level 3 (BSL3).

### Ethics Statement

All of the animal experiments were approved by the Institutional Animal Care and Use Committee (IACUC) of the RML, and performed following the guidelines of the Association for Assessment and Accreditation of Laboratory Animal Care International (AAALAC) by certified staff in an AAALAC-approved facility, following the guidelines and basic principles of the United States Public Health Service Policy on Humane Care and Use of Laboratory Animals and the Guide for the Care and Use of Laboratory Animals.

## Results

### Susceptibility of Various Mouse Strains and Cynomolgus Macaque to Human–Pathogenic Tick-Borne Phlebovirus Infection

In order to compare the susceptibility of various laboratory mouse strains and different age groups of mice to SFTSV infection, we inoculated groups of four mice from nine inbred mouse strains, two recombinant inbred strains, and one outbred strain with a high dose (10^5^ TCID_50_/animal) or a low dose (10^2^ TCID_50_/animal) of SFTSV SD4, i.d., i.p., i.m., or s.c. (**Table 1**). In addition, increased age is a significant risk factor for the severity of disease in SFTS, especially among those aged >60 years (Xiong et al., 2012; Ding et al., 2014; Takahashi et al., 2014), therefore, aged (12–24 months) mouse models based on two different inbred strains (129S1/svlmJ and C57BL6/J), which represented humans aged >60 years (Flurkey et al., 2006), were also infected with SFTSV. None of the mice challenged in this study developed lethal diseases and no significant lesions were found in histological examinations of the mice (**Table 1**). Twelve-month-old C57BL/6 mice and >20-month-old C57BL/6 mice infected with SFTSV via s.c. route, led to only moderate weight loss and all of them survived until 14 dpi.

**Table 1:** Summary of animal models of human-pathogenic tick-borne phleboviruses.

Virus	Animal	Strain	Outcome/Disease	Reference
SFTSV	Mouse	129S1/SvlmJ	Non-lethal	Present study
		129S1/SvlmJ (aged 1 yr)	Non-lethal	Present study
		IFNAR^-/-^ 129/Sv	Lethal	Liu et al., 2014; Shimada et al., 2015
		A/J	Non-lethal	Present study
		BALB/c	Non-lethal	Chen et al., 2012; Jin et al., 2012
		BALB/c (newborn)	Lethal	Chen et al., 2012
		BXD34/TyJ	Non-lethal	Present study
		BXD68/RwwJ	Non-lethal	Present study
		C57BL/6	Non-lethal weight loss	Chen et al., 2012; Jin et al., 2012, Present study
		C57BL/6 (newborn)	Lethal	Chen et al., 2012
		C57BL/6 (aged 1 year)	Non-lethal weight loss	Present study
		C57BL/6 (aged 2 year)	Non-lethal weight loss	Present study
		C57BL/6 (mitomycin C treatment)	Lethal	Jin et al., 2012
		IFNAR^-/-^ C57BL/6	Lethal	Tani et al., 2015, Present study
		CAST/EiJ	Non-lethal	Present study
		CD-1	Non-lethal	Liu et al., 2014, Present study
		CD-1 (newborn)	Non-lethal	Liu et al., 2014
		DBA/1J	Non-lethal	Present study
		DBA/2J	Non-lethal	Present study
		FVB/NJ	Non-lethal	Present study
		Kunming	Non-lethal	Chen et al., 2012
		Kunming (newborn)	Lethal	Chen et al., 2012
		NZBWF1/J	Non-lethal	Present study
		SJL/J	Non-lethal	Present study
	Hamster	Syrian hamster	Non-lethal	Jin et al., 2012
		Golden hamster	Non-lethal	Chen et al., 2012; Liu et al., 2014
		Golden hamster (newborn)	Non-lethal	Chen et al., 2012; Liu et al., 2014
	Rat	Wistar	Non-lethal	Chen et al., 2012
		Wistar (newborn)	Non-lethal	Chen et al., 2012
	Rhesus macaque		Non-lethal	Jin et al., 2015
	Cynomolgus macaque		Non-lethal	Present study
HRTV	Cynomolgus macaque		Non-lethal	Present study

It has been reported that SFTSV infection causes mild illness with thrombocytopenia and leukocytopenia in rhesus macaques. Therefore, in order to investigate whether pathogenic tick-borne phleboviruses induce visible or severe disease in cynomolgus macaques, two groups of two macaques were infected subcutaneously with 10^6^ TCID_50_ of SFTSV SD4 or HRTV Mo4. All four macaques infected with SFTSV or HRTV exhibited no visible clinical signs, except one animal infected with SFTSV had a temporarily decreased platelet count. Moreover, in all four of the infected animals, no viruses were detected from blood during the examination period (at 1, 3, 5, 8, 11, and 14 dpi) and no macroscopic lesions were found at 14 dpi.

### Clinical Disease Signs in IFNAR^-/-^ Mice that Developed Fatal Illness After SFTSV Infection

**FIGURE 1:**
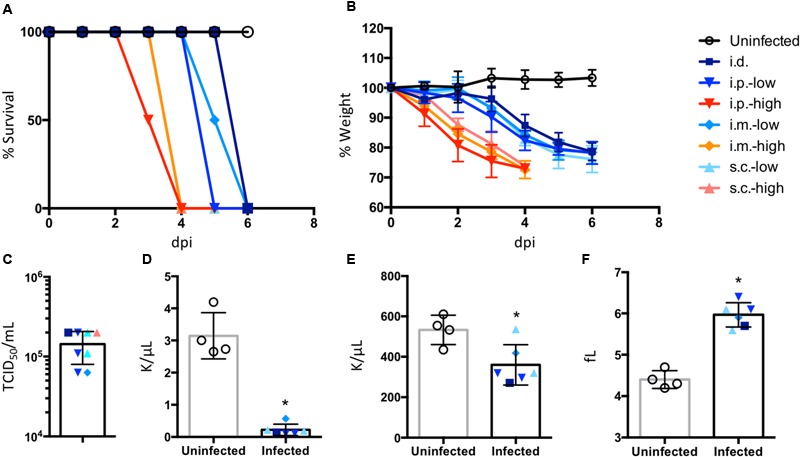
**Clinical manifestations in IFNAR^-/-^ mice infected with SFTSV.** Groups of four IFNAR^-/-^ mice were infected with a high dose (10^5^ TCID_50_/mouse) or low dose (10^2^ TCID_50_/mouse) of the SFTSV strain SD4, intradermally (i.d.), intraperitoneally (i.p.), intramuscularly (i.m.), or subcutaneously (s.c.). The mice were monitored daily to assess survival **(A)** and body weight **(B)** and results until 6 days post inoculation (dpi) were shown. The blood from the infected mice euthanized because they reached the humane endpoint and the uninfected mice euthanized at 6 dpi were subjected to virus titration **(C)** and hematological examinations, i.e., lymphocyte count **(D)**, platelet count **(E)**, and mean platelet volume **(F)**. All values are indicated as means ± SD. ^∗^*P* < 0.05, compared with uninfected controls.

None of the immunocompetent animals tested in this study developed severe/fatal disease after SFTSV infection, so we evaluated the sensitivity and lethality of IFNAR^-/-^ mice via several infection routes and characterized the serum viral load and hematological status of the mice after SFTSV infection. The groups of IFNAR^-/-^ mice inoculated with different doses of SFTSV SD4 *via* different inoculation routes exhibited severe signs of clinical disease, such as severe weight loss, ruﬄed fur, and a hunched posture, and they succumbed to the infection or were euthanized by 6 dpi (**Figure 1**). The average time until death for each group decreased from 6 dpi in the low-dose challenged groups to 4 dpi in the high-dose challenged groups (**Figure 1A**). No significant differences were observed in the survival and weight curves depending on the route of infection (i.e., i.d., i.p., i.m., and s.c.) (**Figures 1A,B**). The MLD_50_ for SFTSV SD4 in IFNAR^-/-^ mice inoculated *via* s.c. was determined as 8.7 × 10^-2^ TCID_50_ (data not shown). The IFNAR^-/-^ mice euthanized at the endpoint of the fatal illness were subjected to virological, hematological, and histopathological examinations in order to investigate the pathogenesis of SFTS in the model. Whole blood samples from eight mice that reached the humane endpoint [i.e., three mice from the i.p.-low (mice inoculated with a low dose of SFTSV *via* i.p.) group, two mice from the s.c.-low group, and one mouse each from the i.d., s.c.-high, and i.m.-low groups] were used in virus titration and those from six out of the eight mice were used in hematological examination. Regardless of the route employed, the infected mice exhibited severe viremia and lymphocytopenia (**Figures 1C,D**). The platelet counts (**Figure 1E**) for the infected mice were significantly lower than those of the control animals, and the mean platelet volumes (**Figure 1F**) of the infected mice were significantly higher, thereby suggesting the occurrence of platelet destruction and the activation of platelet production.

### Pathological and Histopathological Observations of IFNAR^-/-^ Mice Infected with SFTSV

**FIGURE 2:**
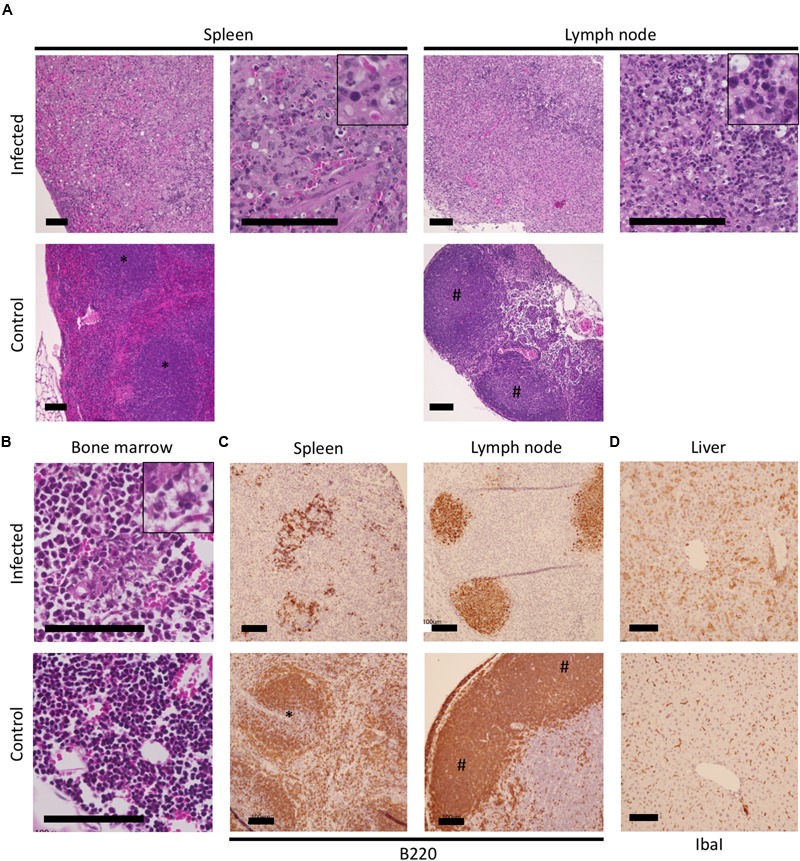
**Histopathological observations of tissues of the IFNAR^-/-^ mice infected with SFTSV.** The spleen, lymph nodes, bone marrow, and livers of SFTSV-infected and uninfected mice were used for histopathologic observations. Losses of the structure with massive apoptosis were observed in hematoxylin and eosin-stained tissues **(A)**; Magnified images of pyknosis and karyorrhexis observed in the necrotic lesions are indicated at the top right corner of the infected spleen and lymph node. The increased myeloid/erythroid ratio in the bone marrow of infected mice was observed **(B)**; A magnified image of moderate necrosis collocating with edema and fibrin is shown at the top right corner. Colocalization of B220-positive cells with necrotic lesions in the spleen and lymph node **(C)** as well as IbaI-positive cells with enlarged cytoplasm in the liver **(D)** were visualized by immunohistochemistry.

Loss of splenic white pulp and diffuse reticuloendothelial hyperplasia of the red pulp were evident in the spleens of infected mice (**Figure 2A**). Destruction of lymphoid follicles was observed in the cortex of the cervical lymph nodes as well. The SFTSV-infected mice developed histiocytic and necrotizing lymphadenitis lesions with pyknosis and karyorrhexis of lymphocytes in the spleen and cervical lymph nodes at the terminal stage of infection (**Figure 2A**, magnified figures). The subcapsular and medullary sinuses frequently contained moderate amounts of edema and fibrin with thrombosis of multiple vessels within the nodes, and histiocytic proliferation was observed in both the medulla and cortex of the affected lymph nodes. In the bone marrow, there was a paucity of erythroid precursor cells and an increase in the myeloid to erythroid ratio (**Figure 2B**). Minimal to moderate necrosis of the bone marrow was collocated with edema and fibrin (**Figure 2B**, magnified figure). B220-positive B cells in the spleen and lymph nodes of the infected mice were apparently decreased in number and distributed in the disrupted structure of each tissue (**Figure 2C**). Furthermore, increased numbers of IbaI-positive cells with enlarged cytoplasm, which might have been Kupfer cells, were observed in the livers of infected mice compared with those in the uninfected mice (**Figure 2D**).

### Target Cells of SFTSV Infection in the Lethal IFNAR^-/-^ Mouse Model

**FIGURE 3:**
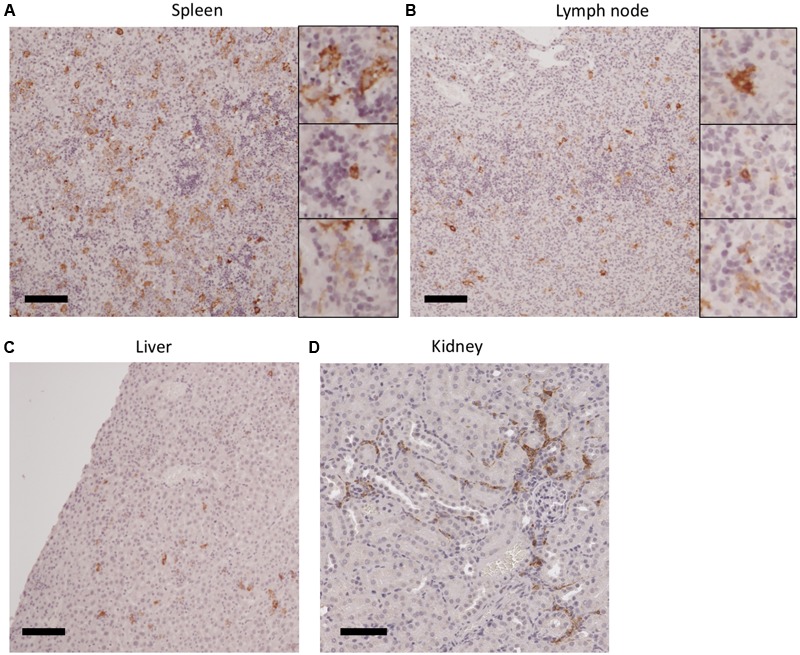
**SFTSV infection in tissues from IFNAR^-/-^ mice infected with SFTSV.** The spleen **(A)**, lymph node **(B)**, liver **(C)**, and kidney **(D)** of SFTSV-infected mice were stained with antibodies raised against SFTSV N antigen. Antigen-positive monocyte-like cells (magnified images at the top), lymphocyte-like cells (middle), and reticular cells (bottom) were morphologically identified in the sample tissues.

Severe fever with thrombocytopenia syndrome virus antigen-positive cells were found in the spleen, lymph nodes, liver, and kidney (**Figure 3**), whereas they were not observed in the spinal cord, gastrointestinal tract, brain, and heart (data not shown). The antigen-positive cells were most abundant in the spleen among the tissues examined. Several antigen-positive cells were morphologically classified into three different cell types (i.e., monocytes, lymphocytes, and reticular cells, magnified figures in **Figure 3**), which were distributed diffusely in the spleen and lymph nodes. However, the necrotic cells in the lesions were rarely positive for the SFTSV antigen. Monocyte-like antigen-positive cells were frequently found surrounding the necrotic lesions in the spleen and lymph nodes. Monocyte-like antigen-positive cells were also observed in the liver but without obvious pathological changes, although the prevalence of the infected cells was clearly lower than that in the spleen and lymph nodes. The interstitial cells of the renal cortex in the kidney were positive for SFTSV antigen. The infected cells in the kidney were distributed sporadically throughout the tissue and they accumulated in the renal capsules and proximal tubules.

**FIGURE 4:**
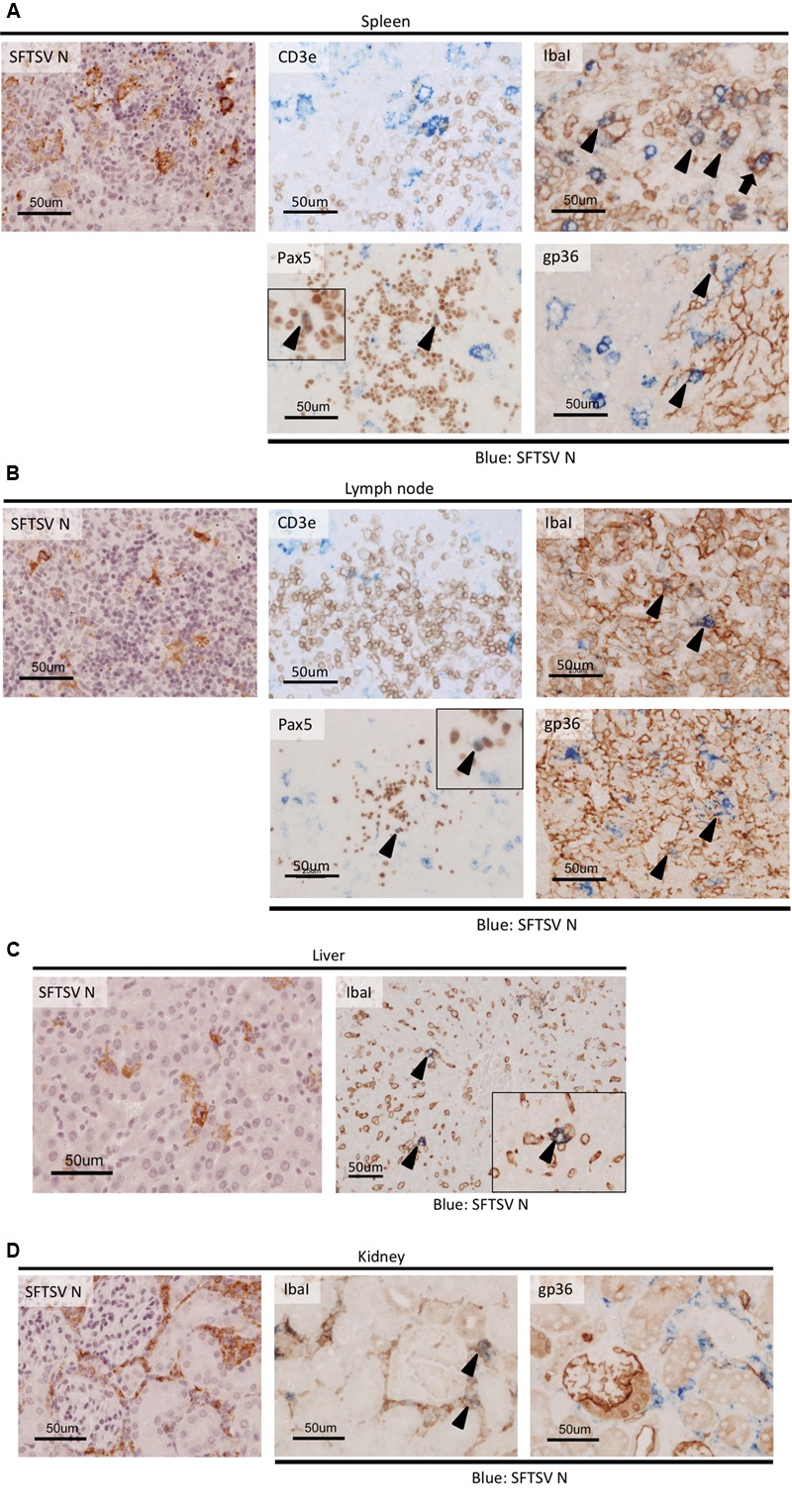
**Colocalization of host cell markers and SFTSV antigen in SFTSV-infected tissues from IFNAR^-/-^ mice.** The spleen **(A)** lymph node **(B)**, liver **(C)**, and kidney **(D)** of the SFTSV-infected mice were subjected to double staining with anti-SFTSV N antibodies and host cellular markers: CD3e (T cells), IbaI (macrophages), Pax5 (immature B cells), and gp36 (reticular cells). Cells stained with both SFTSV and host cellular markers are indicated by arrowheads, and the phagocytosis of an infected cell by an IbaI-positive macrophage is indicated by an arrow.

In order to identify the cells in which the virus replicates, double immunohistochemical staining of the host cell marker(s) and virus antigens was performed using tissues from the spleen, cervical lymph nodes, liver, and kidney (**Figure 4**). In the spleen, the majority of the SFTSV-infected cells overlapped with the IbaI-positive macrophages or gp36-positive reticular cells and none or a few overlapped with the CD3e- or Pax5-positive T or immature B lymphocytes (**Figure 4A**). Phagocytosis of the SFTSV-infected lymphocytes by IbaI-positive macrophages was detected (**Figure 4A**, arrow). IbaI-positive macrophages infected with SFTSV were dispersed throughout the organ. However, while the SFTSV/IbaI double positive cells were major among the SFTSV-infected cells, most of the macrophages in the spleen were not infected with the virus. A limited number of Pax5-positive B cells were found in the same lesions where extensive necrosis of B220-positive B cells (which may have included Pax5-positive cells) was observed in the spleen. Similar overlapping of the SFTSV antigens with IbaI, Pax5, and gp36 was also found in the lymph nodes (**Figure 4B**). There was a high level of overlapping for IbaI and SFTSV antigens in the liver (**Figure 4C**) and kidney (**Figure 4D**). A few interstitial gp36-positive reticular cells in the kidney were infected with SFTSV. Our results demonstrate that macrophages, immature B cells, and reticular cells were infected with SFTSV in the terminal stage of the IFNAR^-/-^ mouse model.

## Discussion

At present, only type I IFN-deficient mouse models [(Liu et al., 2014; Tani et al., 2015), and the present study], newborn mice (Chen et al., 2012), and mitomycin-treated mice (Jin et al., 2012) are available as models for the fatal illness caused by SFTSV infection. Immunocompetent mice [except newborn mice (Chen et al., 2012)], hamsters (Chen et al., 2012; Jin et al., 2012), and macaques (Jin et al., 2015) exhibit moderate disease or are asymptomatic. Therefore, we performed screening of mouse strains with different genetic backgrounds, which contributed to establish a lethal model of monkeypox virus infection (Americo et al., 2010), but none of the immunocompetent mice developed fatal disease with SFTSV infection (**Table 1**). Among the mice challenged in the present study, the two-year-old C57BL/6 mice only exhibited weight loss of not more than 10%, whereas the young mice did not exhibit any weight losses. Thus, factors associated with aging (such as immunological disorders and hematological dysfunctions) may be required to recapitulate the fatal outcome in the mouse model. Several aged mice are usually unavailable for routine investigations, so employing senescence accelerated mice may facilitate the investigation of factors affected by aging over relatively short periods (Vanhooren and Libert, 2013), and this approach should be considered for examining the effects of aging on the fatal outcome.

The present study clearly demonstrated that the IFNAR^-/-^ mouse model recapitulated fatal diseases with hematologic manifestations similar to human cases (i.e., lymphocytopenia and thrombocytopenia). Similar to previous studies with IFNAR^-/-^ (**Table 2**), extensive necrosis and histiocytic proliferation in the lymph nodes were confirmed as the characteristic common histopathological observations in both fatal human cases (Hiraki et al., 2014; Takahashi et al., 2014) and the IFNAR^-/-^ mouse model (Tani et al., 2015). The replication site of SFTSV in the terminal stage of infection was also the lymph nodes in humans and mice. However, hemophagocytosis in the lymph nodes, bone marrow, and spleen, which is a characteristic lesion in fatal human cases, was not observed in our IFNAR^-/-^ mice. In addition, the complete loss of splenic white pulp might be a specific lesion in IFNAR^-/-^ mice. These differences between the mouse model and human cases as well as their genetic background should be considered carefully to explain the pathogenesis of SFTSV infection in mice, especially to discuss the suitability of the model in antiviral or vaccine studies.

**Table 2:** Comparison of histopathological characteristics of SFTSV models and patients.

Model	Time to death	Lymph node	Spleen	Bone marrow	Liver	Kidney	Reference
C57BL/6 IFNAR^-/-^	5–7 days	Histiocytic necrotizing lymphadenitis	Histiocytic necrotizing lymphadenitis	Increasing the myeloid/erythroid ratio	Diffuse infiltration by inflammatory cells	Antigen-positive large mononuclear cells	Tani et al., 2015, Present study
		Loss of lymphoid follicles in the cortex	Loss of white pulp	Moderate necrosis collocating with edema and fibrin	Focal necrosis with slight inflammatory cell infiltration		
					Antigen-positive swollen Kupffer cells		
129/Sv IFNAR^-/-^	2–3 days	Antigen-positive mononuclear cells	Not significant				Liu et al., 2014; Shimada et al., 2015; Hayasaka et al., 2016
C57BL/6	–		Marked increase in megakaryocytes	Significantly increased megakaryocytes	Ballooning degeneration of hepatocytes	Glomerular hypercellularity	Jin et al., 2012; Liu et al., 2014
			Decreased lymphocyte cellularity in the red pulp		Scattered necrosis	Mesangial thickening	
					Multifocal pyknosis, karyorrhexis, and karyokysis	Congestion in Bowman’s space	
Newborn	7–13 days				Large necrotic areas		Chen et al., 2012
					Large amount of mononuclear cells		
Rhesus macaque	–				Multiple scattered loci with hepatocyte necrosis	Glomerular hypercellularity	Jin et al., 2015
						Mesangial thickening	
						Congestion in Bowman’s space	
Human (fatalities)		Necrotizing lymphadenitis with extensive necrosis	Prominent hemophagocytosis	Prominent hemophagocytosis	Mild microvesicular fatty changes	Subepithelial hemorrhage within the renal pelvis	Hiraki et al., 2014; Takahashi et al., 2014; Kim et al., 2016
		Infitration by histiocytes and immunoblasts		Histiocytic hypocellular	Inflammation of lymphocytes and macrophages		
		Prominent hemophagocytosis			Globular necroses and mild portal fibrosis		

The structures of the splenic white pulp and follicles in lymph nodes mainly comprised B cells, so the severe necrotizing lymphadenitis associated with the lesions was probably related to the significant decreases in the white blood cell counts. Furthermore, among the B220-positive B cells in the necrotic lesions, Pax5-positive immature B cells were identified as targets of SFTSV infection. Our results suggest that the immature B cells were: (1) first affected by virus infection in the spleen and lymph nodes, and they then produced cytokines that led to extensive apoptosis and lymphocytopenia; and/or (2) more susceptible to SFTSV infection than the other B cells that remained SFTSV antigen-positive until the terminal stage. The monocytic proliferations near the lesions with massive apoptosis and phagocytosis in the infected cells were antiviral responses by peripheral monocytes that participated in the initial inflammatory response (Serbina et al., 2008), which may also be important for the progression of lymphocyte apoptosis.

Interestingly, the activation of uninfected macrophages was observed throughout the liver, where the infected cells were not detected frequently, in contrast to the case in the spleen or lymph nodes. These macrophages in the liver mainly comprised Kupffer cells (Tani et al., 2015), and thus the activation of hepatic macrophages could be a normal response to the platelets activated by the binding of SFTSV virions (Jin et al., 2012), or a response to infected monocytes permeating from the blood stream. Hepatocytes were not infected with SFTSV in mouse models, but moderate hepatocellular hypertrophy has been found in some IFNAR^-/-^ mice (data not shown) according to a previous report (Liu et al., 2014), which suggests that the cytokines/chemokines produced from the virus-infected Kupffer cells might affect the hepatocytes (Boltjes et al., 2014). In the previous immunocompetent mouse model, proinflammatory cytokines, and chemokines were produced from the liver in SFTSV-infected mice (Sun et al., 2015). In addition, elevated aminotransferases (i.e., AST and ALT) were reported frequently in SFTS patients, even in the recovered cases (Xu et al., 2011; Yu et al., 2011; Sun et al., 2012), thereby indicating that hepatic damage is a common clinical manifestation in humans and currently available animal models. Therefore, Kupffer cells may play a central role in hepatic damage by producing cytokines and chemokines, as well as in thrombocytopenia during SFTSV infection, but the mechanism(s) responsible for Kupffer cell activation remains unclear.

Our histopathological examinations of the mouse model determined the contributions of multiple cell types to pathogenesis in the lethal SFTSV infection as well as the target cells of the virus infection in mice. We demonstrated that this model should be suitable for antiviral drug screening (Tani et al., 2015), or for other purposes that do not require complete immune systems. There were pathological differences between the present study and two previous studies (Liu et al., 2014; Tani et al., 2015) that used IFNAR^-/-^ mice, which were due to the different genetic backgrounds of the mice [129/Sv (Liu et al., 2014) and C57BL/6 (Tani et al., 2015) and the present study)] and/or virus strains [HB29 (Liu et al., 2014), SPL010 (Tani et al., 2015), and SD4 in the present study], as well as the antigen/antibody combinations used for the cellular markers. These differences should be addressed in future research to understand the pathogenesis of SFTS.

## Author Contributions

Conceptualization, KM and HE; Investigation of animal experiments, KM, KM-W, and FF; Investigation of histopathological analysis, YO and DS; Writing – original draft, KM, YO, and DS; Writing – review and editing, YO, DS, FF, ML, HE; Supervision, HE.

## Conflict of Interest Statement

The authors declare that the research was conducted in the absence of any commercial or financial relationships that could be construed as a potential conflict of interest.

## References

[B1] Americo J. L. Moss B. Earl P. L. (2010). Identification of wild-derived inbred mouse strains highly susceptible to monkeypox virus infection for use as small animal models. *J. Virol.* 84 8172–8180. 10.1128/JVI.00621-10 20519404 PMC2916512

[B2] Boltjes A. Movita D. Boonstra A. Woltman A. M. (2014). The role of Kupffer cells in hepatitis B and hepatitis C virus infections. *J. Hepatol.* 61 660–671. 10.1016/j.jhep.2014.04.026 24798624

[B3] Chen X. P. Cong M. L. Li M. H. Kang Y. J. Feng Y. M. Plyusnin A. (2012). Infection and pathogenesis of Huaiyangshan virus (a novel tick-borne bunyavirus) in laboratory rodents. *J. Gen. Virol.* 93 1288–1293. 10.1099/vir.0.041053-0 22357748

[B4] Ding F. Guan X.-H. Kang K. Ding S.-J. Huang L.-Y. Xing X.-S. (2014). Risk factors for bunyavirus-associated severe Fever with thrombocytopenia syndrome, china. *PLoS Negl. Trop. Dis.* 8:e3267 10.1371/journal.pntd.0003267 PMC4199554 25330383

[B5] Flurkey K. Currer J. M. Harrison D. E. (2006). “The mouse in aging research,” in *The Mouse in Biomedical Research* ed. Fox J. G. (Burlington, MA: Elsevier) 637–672.

[B6] Gai Z. T. Zhang Y. Liang M. F. Jin C. Zhang S. Zhu C. B. (2012). Clinical progress and risk factors for death in severe fever with thrombocytopenia syndrome patients. *J. Infect. Dis.* 206 1095–1102. 10.1093/infdis/jis472 22850122

[B7] Hayasaka D. Nishi K. Fuchigami T. Shiogama K. Onouchi T. Shimada S. (2016). 18F-FDG PET imaging for identifying the dynamics of intestinal disease caused by SFTSV infection in a mouse model. *Oncotarget* 7 140–147. 10.18632/oncotarget.6645 26700962 PMC4807988

[B8] Hiraki T. Yoshimitsu M. Suzuki T. Goto Y. Higashi M. Yokoyama S. (2014). Two autopsy cases of severe fever with thrombocytopenia syndrome (SFTS) in Japan: a pathognomonic histological feature and unique complication of SFTS. *Pathol. Int.* 64 569–575. 10.1111/pin.12207 25329676 PMC4282027

[B9] Jin C. Jiang H. Liang M. Han Y. Gu W. Zhang F. (2015). SFTS virus infection in nonhuman primates. *J. Infect. Dis.* 211 915–925. 10.1093/infdis/jiu564 25326554

[B10] Jin C. Liang M. Ning J. Gu W. Jiang H. Wu W. (2012). Pathogenesis of emerging severe fever with thrombocytopenia syndrome virus in C57/BL6 mouse model. *Proc. Natl. Acad. Sci. U.S.A.* 109 10053–10058. 10.1073/pnas.1120246109 22665769 PMC3382536

[B11] Kim N. Kim K.-H. Lee S. J. Park S.-H. Kim I.-S. Lee E. Y. (2016). Bone marrow findings in severe fever with thrombocytopenia syndrome: prominent haemophagocytosis and its implication in haemophagocytic lymphohistiocytosis. *J. Clin. Pathol.* 69 537–541. 10.1136/jclinpath-2015-203417 26908283

[B12] Li D. (2013). A highly pathogenic new bunyavirus emerged in China. *Emerg. Microbes Infect.* 2:e1 10.1038/emi.2013.1 PMC3630492 26038435

[B13] Liu Y. Wu B. Paessler S. Walker D. H. Tesh R. B. Yu X.-J. (2014). The pathogenesis of severe fever with thrombocytopenia syndrome virus infection in alpha/beta interferon knockout mice: insights into the pathologic mechanisms of a new viral hemorrhagic fever. *J. Virol.* 88 1781–1786. 10.1128/JVI.02277-13 24257618 PMC3911604

[B14] Matsuno K. Feldmann H. Ebihara H. (2014). “Severe fever with thrombocytopenia syndrome associated with a novel bunyavirus,” in *Emerging Infectious Diseases* eds Ergonul O. Can F. Akova M. Madoff L. (Amsterdam: Elsevier) 1–12. 10.1016/B978-0-12-416975-3.00001-7

[B15] Matsuno K. Weisend C. Travassos da Rosa A. P. A. Ebihara H. (2013). Characterization of the Bhanja serogroup viruses (Bunyaviridae): a novel species of the genus *Phlebovirus* and its relationship with other emerging tick-borne phleboviruses. *J. Virol.* 87 3719–3728. 10.1128/JVI.02845-12 23325688 PMC3624231

[B16] McMullan L. K. Folk S. M. Kelly A. J. MacNeil A. Goldsmith C. S. Metcalfe M. G. (2012). A new phlebovirus associated with severe febrile illness in Missouri. *N. Engl. J. Med.* 367 834–841. 10.1056/NEJMoa1203378 22931317

[B17] Peng C. Wang H. Zhang W. Zheng X. Tong Q. Jie S. (2016). Decreased monocyte subsets and TLR4-mediated functions in patients with acute severe fever with thrombocytopenia syndrome (SFTS). *Int. J. Infect. Dis.* 43 37–42. 10.1016/j.ijid.2015.12.009 26701820

[B18] Serbina N. V. Jia T. Hohl T. M. Pamer E. G. (2008). Monocyte-mediated defense against microbial pathogens. *Annu. Rev. Immunol.* 26 421–452. 10.1146/annurev.immunol.26.021607.090326 18303997 PMC2921669

[B19] Shimada S. Posadas-Herrera G. Aoki K. Morita K. Hayasaka D. (2015). Therapeutic effect of post-exposure treatment with antiserum on severe fever with thrombocytopenia syndrome (SFTS) in a mouse model of SFTS virus infection. *Virology* 482 19–27. 10.1016/j.virol.2015.03.010 25817401 PMC7125729

[B20] Stubbs A. M. Steele M. T. (2014). Heartland virus disease–United States, 2012-2013. *Ann. Emerg. Med.* 64 314–315. 10.1016/j.annemergmed.2014.06.012 25285350

[B21] Sun Q. Jin C. Zhu L. Liang M. Li C. Cardona C. J. (2015). Host responses and regulation by NFκB signaling in the liver and liver epithelial cells infected with a novel tick-borne bunyavirus. *Sci. Rep.* 5:11816 10.1038/srep11816 PMC4488873 26134299

[B22] Sun Y. Jin C. Zhan F. Wang X. Liang M. Zhang Q. (2012). Host cytokine storm is associated with disease severity of severe fever with thrombocytopenia syndrome. *J. Infect. Dis.* 206 1085–1094. 10.1093/infdis/jis452 22904342

[B23] Takahashi T. Maeda K. Suzuki T. Ishido A. Shigeoka T. Tominaga T. (2014). The first identification and retrospective study of severe fever with thrombocytopenia syndrome in Japan. *J. Infect. Dis.* 209 816–827. 10.1093/infdis/jit603 24231186 PMC7107388

[B24] Tani H. Fukuma A. Fukushi S. Taniguchi S. Yoshikawa T. Iwata-Yoshikawa N. (2015). Efficacy of T-705 (Favipiravir) in the treatment of infections with lethal severe fever with Thrombocytopenia syndrome virus. *mSphere* 1:e61-15. 10.1128/mSphere.00061-15 PMC4863605 27303697

[B25] Vanhooren V. Libert C. (2013). The mouse as a model organism in aging research: usefulness, pitfalls and possibilities. *Ageing Res. Rev.* 12 8–21. 10.1016/j.arr.2012.03.010 22543101

[B26] Xiong W.-Y. Feng Z.-J. Matsui T. Foxwell R. (2012). Risk assessment of human infection with a novel bunyavirus in China. *Western Pac. Surveill. Response J.* 3 69–74. 10.5365/wpsar.2012.3.4.002 23908943 PMC3729083

[B27] Xu B. Liu L. Huang X. Ma H. Zhang Y. Du Y. (2011). Metagenomic analysis of fever, thrombocytopenia and leukopenia syndrome (FTLS) in Henan Province, China: discovery of a new bunyavirus. *PLoS Pathog.* 7:e1002369 10.1371/journal.ppat.1002369 PMC3219706 22114553

[B28] Yu X.-J. Liang M.-F. Zhang S.-Y. Liu Y. Li J.-D. Sun Y.-L. (2011). Fever with thrombocytopenia associated with a novel bunyavirus in China. *N. Engl. J. Med.* 364 1523–1532. 10.1056/NEJMoa1010095 21410387 PMC3113718

[B29] Yun S.-M. Song B. G. Choi W. Roh J. Y. Lee Y.-J. Park W. I. (2016). First isolation of severe fever with thrombocytopenia syndrome virus from haemaphysalis longicornis ticks collected in severe fever with thrombocytopenia syndrome outbreak areas in the republic of Korea. *Vector Borne Zoonotic Dis.* 16 66–70. 10.1089/vbz.2015.1832 26745758 PMC4742983

